# Methodologic considerations in estimating racial disparity of mortality among very preterm infants

**DOI:** 10.1038/s41390-024-03485-w

**Published:** 2024-08-23

**Authors:** Siyuan Jiang, Laura A. Rose, Jeffrey B. Gould, Mihoko V. Bennett, Jochen Profit, Henry C. Lee

**Affiliations:** 1 Division of Neonatology, University of California San Diego, La Jolla, CA USA; 2 Division of Neonatology, Children’s Hospital of Fudan University, Shanghai, China; 3 Division of Neonatology, Stanford University, Stanford, CA USA; 4 California Perinatal Quality Care Collaborative, Stanford University, Stanford, CA USA

## Abstract

**Abstract:**

This review explores methodological considerations in estimating racial disparities in mortality among very preterm infants (VPIs). Significant methodological variations are evident across studies, potentially affecting the estimated mortality rates of VPIs across racial groups and influencing the perceived direction and magnitude of racial disparities. Key methodological approaches include the birth-based approach versus the fetuses-at-risk approach, with each offering distinct insights depending on the specific research questions posed. Cohort selection and the decision for crude versus adjusted comparison are also critical elements that shape the outcomes and interpretations of these studies. This review underscores the importance of careful methodological planning and highlights that no single approach is definitively superior; rather, each has its strengths and limitations depending on the research objectives. The findings suggest that adjusting the methodological approach to align with specific research questions and contexts is essential for accurately assessing and addressing racial disparities in neonatal mortality.

**Impact:**

- Elucidates the impact of methodological choices on perceived racial disparities in neonatal mortality.
- Offers a comprehensive comparison of birth-based vs. fetuses-at-risk approaches in the context of racial disparity research.
- Provides guidance on the cohort selection and adjustment criteria critical for interpreting studies on racial disparities in very preterm infant mortality.

## Introduction

Racial and ethnic disparities in newborn health outcomes arise from a complex interplay of socioeconomic and healthcare-related factors across different levels.^1–3^ Maternal health status, fetal and neonatal factors, hospital resources, provider attitudes, and social and neighborhood environments all contribute significantly to these disparities. Accurately measuring these disparities is challenging and heavily influenced by the methodological choices made in study design, which can significantly impact the findings.^1–5^ Currently, there is variability in methodological approaches used to estimate racial disparities in preterm infant mortality, with the chosen approach potentially altering the observed direction and extent of disparities.^1^ Selection of the source population also has implications on mortality rates and requires careful consideration. In this review, we highlight differences in common methodological approaches to estimating racial disparities in mortality of very preterm infants and discuss how particular methods might be suitable for various research goals. We consider the very preterm infant (VPI) as those born prior to 32 weeks gestational age and the extremely preterm infant (EPI) as those born prior to 28 weeks.

## Birth-based approach vs. fetuses-at-risk approach

The examination of racial disparities in VPI mortality is underpinned by two methodological approaches: the birth-based approach and the fetuses-at-risk (FAR) approach.^6–10^ The birth-based method calculates mortality rates exclusively based on births, which are predominantly live births. In contrast, the FAR approach considers all fetuses beyond a specified gestational age as at risk.^6^ This distinction has led to seemingly contradictory findings. Table 1 shows results from several recent studies using US national vital statistics data: the birth-based approach typically shows lower mortality rates among Black VPIs/EPIs compared to White infants, with a rate ratio approximately between 0.8–0.9; while under the FAR approach, this trend reverses, revealing Black very preterm or extremely preterm fetuses experiencing 2–3 times the mortality rate of White fetuses.^8–10^

**Table 1:** Racial disparity of neonatal mortality among very preterm infants estimated using birth-based vs. fetuses-at-risk approaches.

References	Data source	Year	Population	Types of mortality	Birth-based approach (Black vs. White)	Fetuses-at-risk approach (/1000 fetus-at-risk) (Black vs. White)
Wu, JAMA Pediatr, 2023^8^	US National Center for Health Statistics	2014–2018	22–41 weeks, Liveborn singleton, excluding CA	Neonatal death ( < 28 days of life)	22–27w: 23.1% vs. 25.6% 28–31w: 2.5% vs. 3.3%	22–27w: 2.4 vs. 0.86 28–31w: 0.35 vs. 0.19
Shukla, J Perinatol, 2024^10^	CDC WONDER	2007–2018	22–42 weeks, Liveborn, excluding CA	Neonatal death ( < 28 days of life)	22w: 68.8% vs. 73.3%; RR: 0.94 (0.92–0.95) 23w: 45.0% vs. 53.5%; RR: 0.84 (0.82–0.86) 24w: 24.6% vs. 30.1%; RR: 0.82 (0.79–0.84) 25w: 14.8% vs. 17.6%; RR: 0.84 (0.80–0.87) 26w: 9.7% vs. 11.1%; RR: 0.87 (0.83–0.92) 27w: 6.2% vs. 6.8%; RR: 0.91 (0.85–0.96) 28w: 3.8% vs. 3.8%; RR: 1.01 (0.94–1.08) 29w: 2.5% vs. 2.4%; RR: 1.03 (0.95–1.11) 30w: 1.6% vs. 1.5%; RR: 1.03 (0.94–1.12) 31w: 1.1% vs. 1.0%; RR: 1.07 (0.97–1.18)	22w: 0.94 vs. 0.30; RR: 3.08 (2.98–3.17) 23w: 0.77 vs. 0.29; RR: 2.62 (2.54–2.71) 24w: 0.56 vs. 0.23; RR: 2.42 (2.33–2.51) 25w: 0.38 vs. 0.16; RR: 2.38 (2.27–2.49) 26w: 0.28 vs. 0.12; RR: 2.36 (2.24–2.49) 27w: 0.20 vs. 0.09; RR: 2.28 (2.14–2.43) 28w: 0.15 vs. 0.07; RR: 2.22 (2.06–2.38) 29w: 0.11 vs. 0.05; RR: 2.12 (1.95–2.30) 30w: 0.09 vs. 0.05; RR: 1.98 (1.81–2.16) 31w: 0.08 vs. 0.04; RR: 1.95 (1.77–2.15)
Botin, Plos One, 2021^9^	US National Center for Health Statistics	2006–2015	≥24 weeks, Liveborn singleton, excluding CA	Early neonatal death (within 7 days of life)	24–31w: 4.7% vs. 5.5%; RR: 0.86 (0.84–0.89)	24–31w: 1.01 vs. 0.48; RR: 2.11 (2.05–2.18)

*CA* congenital anomaly. *RR* risk ratio.

**Fig. 1:**
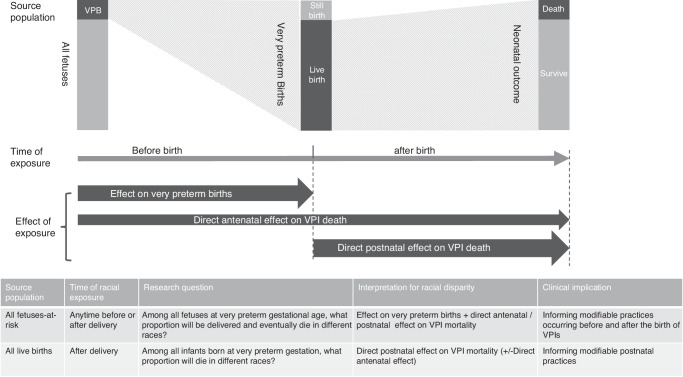
Fetuses-at-risk vs. birth-based approaches in evaluating racial disparities in mortality among very preterm infants. This figure highlights the fundamental distinctions between the fetuses-at-risk approach and the birth-based approach, focusing on differences in source populations and the timing of racial exposure. These distinctions define how each approach addresses unique research questions, illustrating that the racial disparities observed by each method reveal different facets of the impact of racial exposure on mortality among very preterm infants.

This divergence raises controversy regarding which approach more effectively illuminates the racial disparities in VPI outcomes.^7,11,12^ Both the birth-based and FAR approaches contribute valuable perspectives, with the choice between them hinging on the specific research questions and aspects of care under consideration. The FAR approach, by including a broader at-risk population, allows for an analysis of the total impact of racial and ethnic disparities on VPI outcomes. This includes the racial influence of very preterm birth rates and the direct racial effects on VPI outcomes, spanning both before and after birth (Fig. 1).^7^ The comprehensive nature of disparities uncovered by the FAR method may carry broader implications for public health and obstetric practices. However, the results of this approach are highly driven by the differences in very or extremely preterm birth rates, so it may not be suitable to capture the nuances of postnatal care practices’ impact.

Conversely, the birth-based approach provides a more focused lens on the postnatal period, examining the direct influence of racial factors on the outcomes of infants who have been born. This specificity makes it especially relevant to neonatal healthcare professionals. By offering a targeted view of racial disparities that emerge after birth, the birth-based method underscores the importance of postnatal care in addressing these disparities.^2,13,14^

Therefore, the decision to employ the birth-based versus FAR approach should be informed by the research aim and the domain of care being investigated. Recognizing the distinct advantages and limitations of each method is essential for a thorough understanding of racial disparities in VPI mortality. Based on the current data, it appears that during the very preterm period, because 2 to 3 times as many Black than white fetuses will be born during the very preterm period, Black fetuses face a 2–3 times higher risk of mortality compared to white fetuses; however, postnatally, among liveborn VPIs, Black infants exhibit a lower risk of death compared with white infants.^8–10^ The exact reasons for the relatively low mortality risk among Black VPIs require further investigation. This postnatal “advantage” for Black infants may partly be a reflection of the disproportionately high number of Black VPIs^1^. It should be kept in mind that despite the relative risk, the absolute number of Black VPIs that die is still higher than that of white VPIs in almost all contexts in the United States. In addition to mortality, morbidities such as bronchopulmonary dysplasia also show conflicting results under different approaches. Bronchopulmonary dysplasia seems to be less frequently observed in Black VPIs when using the birth-based approach.^15–17^ However, the FAR approach yields differing estimates of risk showing disadvantage rather than advantage for Black infants.^7^

## Cohort selection

**Fig. 2:**
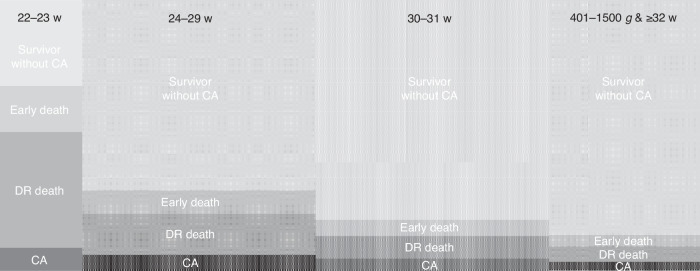
Variations in cohort selection for evaluating racial disparities in mortality among very preterm infants. The main variations in cohort selection include gestational age and birth weight criteria, and the inclusion and exclusion of early deaths, delivery room deaths, and congenital anomalies (Table 2). The distribution of infants is not uniform across different gestational age or birth weight categories. Additionally, the incidence rates of neonatal death, early or delivery room death, and congenital anomalies vary significantly among these groups. Therefore, the cohort selection criteria can profoundly influence the estimated mortality rates and the perceived racial disparities in mortality among very preterm infants.

**Fig. 3:**
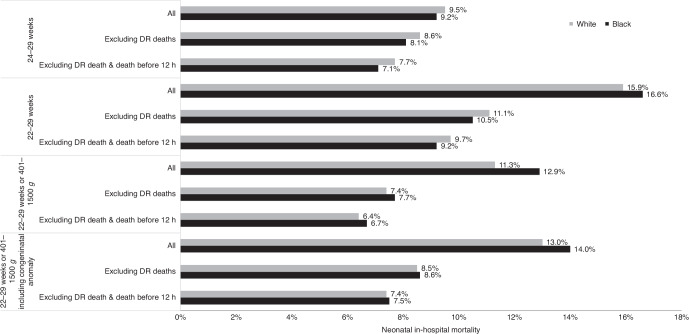
Impact of cohort selection on estimating racial disparities in neonatal mortality among extremely preterm infants. Data from reference.^1^ DR delivery room.

In studies examining mortality among very or extremely live preterm infants, the process of cohort selection (inclusion and exclusion) also introduces variations that influence the results (Table 2, Fig. 2).^1,2,13,14,18,19^ A notable example is a comprehensive assessment using population-based data from California, which highlighted that cohort selection could lead to nearly a threefold difference in estimated mortality rates of EPIs (Fig. 3).^1^ Studies employing various inclusion and exclusion criteria also found varying extent and direction of racial disparities (Table 2). The key aspects influencing cohort selection and their implications are as follows:

**Table 2:** Cohort selection and crude vs. adjusted estimation of racial disparity of neonatal mortality among very preterm infants.

Data source	Study Year	Population/ Cohort selection			Outcome	Crude comparison (Black vs. White)	Adjusted comparison (Black vs. White)	
		GA/BW	Inclusion	Exclusion			Adjusted factors	Adjusted results
US National Center for Health Statistics	1995–2020	22–31 weeks	Liveborn	/	Infant death (0–364 days)	2018–2020 22w: 69.0% vs. 76.8%; RR: 0.90 (0.81–0.99) 23w: 44.3% vs. 55.9%; RR: 0.79 (0.72–0.87) 24w: 25.7% vs. 35.8%; RR: 0.72 (0.65–0.79) 25w: 19.0% vs. 22.1%; RR: 0.86 (0.78–0.95) 26w: 12.0% vs. 15.4%; RR: 0.78 (0.69–0.87) 27w: 8.7% vs. 10.4%; RR: 0.84 (0.74–0.95) 22–27w: 23.8% vs. 27.2%; RR 0.87 (0.84–0.91) 28–31w: 3.8% vs. 3.9%; RR 0.99 (0.93–1.06)	/	/
VON, 789 NICUs in US or Puerto Rico	2006–2017	22–29 weeks	Liveborn singleton	Congenital anomalies	In-hospital death	18.6% vs. 16.4%	/	/
NICHD-NRN, 25 NICUs in US	2002–2016	22–27 weeks and 401–1500 g	Live inborn	Infants with congenital anomalies who were not resuscitated	In-hospital death	2002: 35% vs. 30% 2016: 24% vs. 22%	**Infant biological variables:** GA, BW, sex, multiple gestation, SGA **Maternal biological variables:** diabetes, hypertension, antepartum hemorrhage **Care practices:** antenatal steroids, mode of delivery, birth center, prenatal care, center **Socioeconomic factors:** maternal age, educational level, insurance status, marital status	Adjusted mortality 2002: 16% vs. 21% 2016: 12% vs. 15%
CPQCC	2014–2018	22–29 weeks	Liveborn	Congenital anomaly	In-hospital death	16.6% vs. 15.9%; RR: 1.04 (0.92–1.17)	**Biologic variables:** GA, multiple gestation, sex, SGA **Potentially modifiable risk factors:** maternal age, maternal health conditions, maternal care factors (prenatal care, cesarean delivery, antenatal steroids, location of birth), Apgar score at 5 min	aRR: 0.84 (0.76–0.93)
CPQCC	2010–2014	25–29 weeks or 401–1500 g	Liveborn	Delivery room death, death <12 h, congenital anomalies	In-hospital death	4% vs. 4% (not significant)	/	/
Population-based cohort in 39 New York hospitals	2010–2014	24–31 weeks	Liveborn	Congenital anomalies	Neonatal death ( < 28 days) or in-hospital death up to 1 year	8.4% vs. 7.2% (not significant)	**Maternal sociodemographic characteristics:** age, BMI, educational level, insurance, nativity, prenatal visits, singleton, parity, smoke, alcohol, drugs, maternal complications, type of delivery **Infant factors occurring before birth:** GA, z-score, sex, Apgar at 1 min	aOR 1.21 (0.87–1.68)

*GA* gestational age, *BW* birth weight, *SGA* small for gestational age, *VON* Vermont Oxford Network, *NICHD-NRN* Eunice Kennedy Shriver National Institute of Child Health and Human Development Neonatal Research Network, *CPQCC* California Perinatal Quality Care Collaborative.

### Lower gestational age cut-off

The practice of initiating active treatment for infants at 22–23 weeks’ gestation varies across hospitals and regions, leading to inconsistent inclusion of these infants in research. Their notably high mortality rates mean that their inclusion or exclusion can significantly affect mortality estimates. For instance, overall mortality estimates, including delivery room and early deaths, vary significantly between cohorts with lower gestational age cut-offs at 22 weeks versus those starting at 24 weeks (16% vs. 9%, Fig. 3).^1^ It is also worth noting that the analysis of racial disparities shows a potential reversal in the direction of absolute mortality difference when extending the lower gestational age cut-off from 24 to 22 weeks, albeit without statistical significance. Such reversal does not imply that Black infants at 22–23 weeks have higher mortality than White infants. On the contrary, Black infants at 22–23 weeks exhibit significantly lower mortality compared to white infants, but the higher proportion of birth at 22–23 weeks among Black EPIs causes this reversal.

### Upper gestational age cut-off

The upper gestational age cut-off also varied from 27 to 31 weeks across studies (Table 2). Expanding the upper gestational age limit can dilute the representation of overall mortality for infants with smaller gestational age. Moreover, racial effects also differ across gestational age groups. The seeming survival advantage for Black infants diminishes and eventually reverses in favor of white infants around 32–34 weeks.^18^ Thus, varying the upper gestational age cut-off can alter the perceived direction and magnitude of racial disparities.

### Birth weight criteria

Birth weight criteria have also been applied parallel or serial to gestational age. Compared with serial criteria (“and”) of gestational age and birth weight, the parallel criteria (“or”) generate more significant effect on mortality. Specifically, including infants with a birth weight of 401–1500 g alongside those at 22–29 weeks generally results in the inclusion of infants with a gestational age ≥ 30 weeks, affecting mortality similarly to adjustments in the upper gestational age cut-off. As shown in Fig. 3, the overall mortality decreases from 16 to 12% after adding the birthweight criteria.^1^ What’s more, after applying birth weight criteria, there is a trend toward an increase in the overall mortality disparity between Black and white infants, with the direction of differences in mortality, excluding early death, being reversed. This change is primarily due to the higher mortality among Black infants with a birth weight of 401–1500 g and gestational age ≥ 30 weeks. The relatively lower proportion of Black infants with a higher gestational age further contributes to the increase in disparity.

### Exclusion of delivery room and early neonatal death

Several considerations must be weighed when deciding whether to exclude early neonatal deaths from analyses. Firstly, delivery room or early neonatal deaths are closely linked with the status at birth, decisions regarding active care, and delivery room management practices. Therefore, excluding these early deaths from analyses makes the resulting population more suitable for evaluating racial disparities influenced by variations in practices post-NICU admission. Secondly, early neonatal mortality is disproportionately high among infants at 22–23 weeks (Fig. 2); excluding these early deaths can lead to a significant reduction in the overall mortality estimation.^1,20^ For instance, with a 46% delivery room mortality rate for infants born at 22–23 weeks, excluding delivery room deaths resulted in a one-third reduction of the overall mortality from 16.1% to 10.9% in California (Fig. 3).^1^ Thirdly, in cases where universal active resuscitation is provided, such as ≥ 24 or 25 weeks, both the absolute delivery room mortality and the proportion of delivery room deaths relative to overall deaths are relatively low. This suggests that the inclusion or exclusion of these deaths may not significantly affect the extent or direction of comparisons across different groups, such as by location or race.^1,21^ Lastly, potential racial disparities in the rates of active management and death rates in the delivery room for periviable infants may offer additional insights into the impact of racial factors on perinatal decision making, and deserve targeted and independent investigation.^1,22,23^

### Exclusion of congenital anomaly

Most studies investigating racial disparities exclude infants with congenital anomalies (Table 2). This selection strategy aims to create a “healthier” preterm population that allows for a more unambiguous evaluation of the association between postnatal exposures and outcomes, by minimizing confounding from potentially varying rates and severities of congenital anomalies across different exposure groups. Very or extremely preterm infants generally have a higher incidence of congenital anomalies with high mortality. For example, in California, 6.7% of infants at 22–29 weeks’ gestation or weighing 401–1500 g were noted to have congenital anomalies, with an overall mortality of 17.5%.^1^ Consequently, the exclusion of infants with congenital anomalies resulted in a 12% reduction in the absolute overall mortality rate, from 13.6% to 12.0% (Fig. 3). However, the exclusion of infants with congenital anomaly did not appear to significantly affect the direction and extent of racial disparity (Fig. 3). Notably, Black infants exhibited a lower incidence of congenital anomaly (5.1% vs. 7.3%) compared to white infants, but showed a significantly higher mortality (34.5% vs. 14.1%) from such conditions. This discrepancy may be explained by increased severity of congenital abnormalities in Black compared to White infants, differences in maternal risk status such as comorbidities that also impact neonatal outcomes, or racial differences in treatment for congenital anomalies.^24^ For example, the Black: White mortality disparity among infants with congenital heart disease was found to be wider in the post-neonatal period than the neonatal period, even after adjusting for maternal risk status, implying that timing of surgical procedures may differ in Black compared with white infants.^25^ The differential distribution and outcomes of infants with congenital anomalies across racial groups may necessitate independent investigation and verification to fully understand the implication of these findings.

In summary, cohort selection is another important methodology issue in shaping the outcomes and interpretations of research into racial disparities in VPI mortality. Variations in selection criteria may alter perceptions of the disparities’ magnitude and direction. Therefore, it is essential to exercise meticulous care in the cohort selection process, ensuring the study population accurately represents the source population and is aptly chosen to address the research question at hand.

## Crude vs. adjusted comparisons

Risk adjustments are frequently applied in studies evaluating racial disparity of VPI mortality, while variations exist in the adoption of adjustments and the selection of adjustment variables. Results should be interpreted in context of the specific research question, with attention to appropriateness of the risk adjustment strategy.

### To adjust or not to adjust

**Fig. 4:**
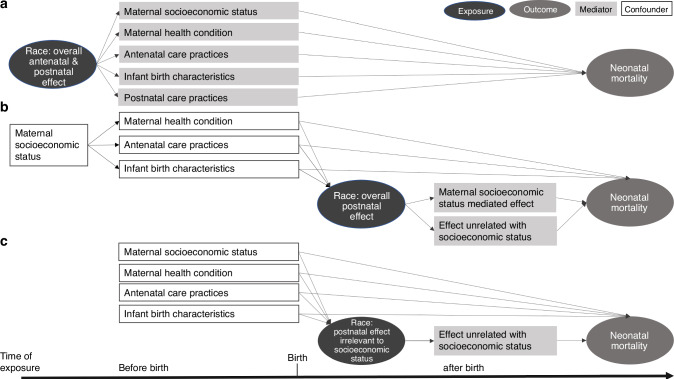
Different adjusting factors in evaluating racial disparities in mortality among very preterm infants. This figure maps out the different adjusting factors considered when evaluating the racial disparities in neonatal mortality. **a** Evaluating the overall antenatal and postnatal effects of racial exposure on neonatal mortality: In this scenario, maternal socioeconomic status, health conditions, antenatal care, infant birth characteristics, and postnatal care are viewed as mediators in the causal pathway from race to neonatal mortality. **b** Evaluating the overall postnatal effect of race on neonatal mortality: In this scenario, antenatal factors such as maternal health condition, antenatal care and infant birth characteristics act as confounders. The postnatal effect of race could be mediated by, or independent of, maternal social economic status. **c** Evaluating the postnatal effect of race on neonatal mortality independent of maternal socioeconomic status: In this scenario, maternal socioeconomic status is treated as a confounder. The analysis seeks to isolate the postnatal effects of race on neonatal mortality from the influences of socioeconomic factors.

Most studies adjust for factors including maternal socioeconomic status, maternal health condition, antenatal care practices, and infant birth characteristics (Table 2). Some investigators argue that adjusting for such variables related to medical care will mask the effects of race as well as related factors and therefore mask disparities.^13^ The purpose of adjustment is to identify the independent association between race and neonatal mortality, by controlling for other variables that could confound the association. So, the decision to adjust is determined by what race represents in a specific study context. In studies assessing the overall antenatal and postnatal effect of race on neonatal mortality, all the aforementioned factors may lie within the causal pathway from race to neonatal mortality (Fig. 4a), and act as mediators; thus, the adjustment of these factors could diminish the race-mortality association. Conversely, in studies focusing on postnatal or neonatal periods, race serves as a proxy for the impact of postnatal care practice (Fig. 4b, c). Factors occurring before and at birth may influence both the exposure (racial-related postnatal care practice) and the outcome and may be adjusted as confounders. The interpretation of such analysis assumes that any adjusted racial effect on neonatal mortality is due to postnatal or post-admission events.

There is often a contradiction between unadjusted and adjusted associations between race and VPI mortality. Despite higher or similar mortality of observed mortality among the Black VPI/EPIs, the adjusted analyses show better outcomes among Black VPI/ EPIs compared to white infants.^1,2^ This may indicate that factors such as gestational age and clinical status at birth have the most significant impact on the observed racial disparity.^1^ Some of these factors may be outside the direct control of healthcare providers, while others may depend on the approach of the combined obstetric and neonatal teams. Mortality by gestational age stratification also replicates this finding.^18^ Successful interventions aimed at addressing social determinants of health to improve maternal and neonatal health outcomes would decrease the discrepancy between unadjusted and adjusted analyses.

### What to adjust

There are also variations in the factors adjusted for when evaluating the postnatal association between race and neonatal mortality (Table 2). A common point of contention is whether to adjust for maternal socioeconomic status. Again, the decision to include these factors in multivariable models depends on the specific research question. The postnatal racial effect may arise from two sources: effects mediated by socioeconomic status and those unrelated to socioeconomic status (Fig. 4b). For example, racial disparities in breast milk or human milk feeding for VPIs may be mediated by socioeconomic factors, which influence cultural norms, parental leave policies, visitation opportunities, and availability of lactation support.^26,27^ In addition to these factors, hospital practices and quality improvement efforts can also significantly influence the provision of breast milk for VPIs.^28^ Therefore, when the objective is to evaluate the overall postnatal racial effect, maternal socioeconomic status may have mediation effect and generally should not be adjusted. In contrast, when the study aims to ascertain racial disparity irrespective of socioeconomic status, this status acts as a confounder (Fig. 4c). This approach may have significance in guiding quality improvement in NICUs, as effects unrelated to socioeconomic status may be more closely related with NICU care practice and could be modified in the NICU environment.

Another decision point in methodology is adjustment for treating hospitals. Both between-hospital and within-hospital racial variation exist in care practice and outcomes for VPIs.^14,19^ When centers are not accounted for in adjustment, the association between race and neonatal mortality reflect the combined effect of both between- and within- hospital care variations. For example, Black and Hispanic infants may be more likely to receive care in NICUs with worse quality measures. They may also receive lower quality of care compared to white infants within the same NICU. When adjusting for the hospital as a cluster, the between-NICU disparity may be obscured, and the associations observed may reflect more the effect of within-hospital practice variations. In situations where a hospital or group of hospitals do not provide optimal care to Black infants or other minoritized populations, incorporating the hospital as an adjustment factor may mask such disparities.

## Conclusion

In conclusion, significant methodological variations exist in studies that assess racial disparities in mortality among VPIs. These methodological differences can substantially affect the estimated mortality rates of VPIs across different racial groups and potentially alter the perceived direction and magnitude of racial disparities. There is no singular “best” or “correct” methodology for studying racial disparities; each approach offers valuable insights and implications. The selection of the source population, as well as the careful definition of inclusion and exclusion criteria and the choice of analysis model, requires meticulous consideration. These decisions must align closely with the specific objectives of the research question to ensure that the findings are both robust and relevant.
